# Root Biomass Distribution of *Populus sibirica* and *Ulmus pumila* Afforestation Stands Is Affected by Watering Regimes and Fertilization in the Mongolian Semi-arid Steppe

**DOI:** 10.3389/fpls.2021.638828

**Published:** 2021-04-23

**Authors:** Batkhuu Nyam-Osor, Ser-Oddamba Byambadorj, Byung Bae Park, Mattia Terzaghi, Gabriella Stefania Scippa, John A. Stanturf, Donato Chiatante, Antonio Montagnoli

**Affiliations:** ^1^Laboratory of Forest Genetics and Ecophysiology, School of Engineering and Applied Sciences, National University of Mongolia, Ulaanbaatar, Mongolia; ^2^Laboratory of Silviculture, College of Agriculture and Life Science, Chungnam National University, Deajeon, South Korea; ^3^Department of Chemistry and Biology “A. Zambelli”, University of Salerno, Salerno, Italy; ^4^Department of Biosciences and Territory, University of Molise, Contrada Fonte Lappone, Isernia, Italy; ^5^Institute of Forestry and Rural Engineering, Estonian University of Life Sciences, Tartu, Estonia; ^6^Laboratory of Environmental and Applied Botany, Department of Biotechnology and Life Science, University of Insubria, Varese, Italy

**Keywords:** siberian poplar, siberian elm, land degradation and desertification, forest shelterbelt, restoration, tree roots

## Abstract

Desertification of the semi-arid steppe of Mongolia is advancing very rapidly, motivating afforestation efforts. The “Green Belt” joint project (Government of Mongolia and Republic of Korea), which aims to mitigate soil degradation and develop agroforestry activities through the planting of a forest shelterbelt, is one such response. In these plantations, tree growth has been supported by different watering regimes (no watering, 2, 4, and 8 L h^−1^) and by two types of soil fertilization (NPK and Compost). The present paper analyses the effect of these techniques on soil chemistry and root biomass partitioning of *Populus sibirica* (Horth ex Tausch) and *Ulmus pumila* (L.) tree species. In July 2019, at the plantation site in Lun Soum, Tuv province (Mongolia), six trees were excavated by hand in each treatment, the root system was divided into taproot and five diameter classes (0–2; 2–5; 5–10; 10–20; > 20 mm), and the biomass was measured. Soil organic matter, macronutrients, and pH were also measured. The addition of fertilizers in the long-term did not enhance the soil chemical properties. The build-up of root biomass in both species correlated positively with increasing levels of the watering, while the application of fertilizers led to root growth suppression. For most of the root classes and both species, an irrigation level of 4 L h^−1^ was sufficient to yield the highest biomass and could be recommended for afforesting the semi-arid steppe of Mongolia. The root biomass of *P. sibirica* was more dependent on the watering regimes and of *U. pumila* was more negatively influenced by the application of fertilizers, indicating that *U. pumila*, due to the its lower water need, could be suitable for afforesting semi-arid environments. Our experiments suggest that afforestation practices in the semi-arid steppe of Mongolia should be supported by a prior analysis of plants' needs, soil type, dose, and type of fertilizers to be applied. Knowledge of the root response to the supporting techniques is necessary for choosing the best one for the plantation and, thus, to develop a sustainable and successful strategy to restore these degraded lands.

## Introduction

A considerable percentage (78%) of Mongolian arid and semi-arid lands are highly degraded and prone to desertification (Tsogtbaatar, 2004, 2009). To the aim of counteracting soil degradation and promoting agroforestry activities, in 2008 the Mongolian and South Korean governments jointly promoted a forested shelterbelt in the Mongolian arid and semi-arid lands named the “Green Belt” Project (Lee and Ahn, 2016; Byambadorj et al., 2020). The establishment of a new tree plantation in these lands represents a difficult task especially when extreme periods of cold and heat waves overlie the constant condition of water shortage. The interplay of these adverse environmental factors may explain why afforestation and reforestation efforts often are unsuccessful (Choi, 2004; Cao, 2008; Wang et al., 2010). Therefore, a careful selection of both plant material and management techniques is necessary to support adequate survival and rapid development of the planted trees, ensuring the success of the restoration interventions (FAO, 2004; Mansourian et al., 2005; Thomas et al., 2014). In particular, the tree species selection must take into consideration their current suitability and adaptability as well as the resistance to future environmental conditions (Ffolliott et al., 1995; Meli et al., 2014; Lu et al., 2017). However, the selection of tree species is also crucial for providing ecosystem services (Gamfeldt et al., 2013; Reisman-Berman et al., 2019). Indeed, forests provide a range of functions that are fundamental to sustaining terrestrial systems through the regeneration of soil and biodiversity, water conservation, groundwater recharge, dust and flood prevention (MEA., 2005; Chazdon et al., 2009; Abson et al., 2014). Also, forests directly provide vital provisioning services such as biomass for materials and energy (Vitousek et al., 1986; Rojstaczer et al., 2001; Felton et al., 2020), food, recreation, and sheltering areas (Mander et al., 2007; Nassauer and Opdam, 2008; Scherr and McNeely, 2008; Lovell and Johnston, 2009; Chirwa and Mahamane, 2017). Thus, a selection of tree species that can be used in afforestation of dryland areas should also aim to provide at the best the above-mentioned ecosystem services, taking into account other biological characteristics (Reisman-Berman et al., 2019).

The implementation of management techniques is also of fundamental importance for afforestation (Siyag, 2014; Zhang et al., 2016). In nutrient-deficit soils when plant productivity is one of the main objectives, fertilization is considered essential (FAO Soil Portal, Management of some problem soils; Marschner, 1995; Siyag, 2014). Fertilization with inorganic materials is the main practice, but organic manures also can supply nutrients in slowly available forms and improve soil physicochemical properties. The best approach is to use a mixture that includes macro- and micro-nutrients (Gregory, 2008; Giehl et al., 2013). However, the most commonly used fertilization mixtures include macronutrients such as nitrogen (N), phosphorous (P), and potassium (K). Furthermore, in semi-arid environments, the success of afforestation measures will strongly depend on the adoption of an adequate watering regime. It is well-known that watering regimes affect water-use characteristics (Li et al., 2013), water consumption (Gao et al., 2011), and protein content in plants due to the variations of water availability in the soil. Yao et al. (2016) have reported that afforestation of semi-arid or arid lands could lead to variations of the soil moisture content in the upper 30 cm of soil. The regime adopted depends on the water available on site and the chemical, and physical nature of the soil. Indeed, soil texture, structure, drainage and solid material composition (mineral and organic) affects its water-storing capabilities, drainage and evaporation (Bonsu, 1997; Scherer et al., 2013; BIO Intelligence Service., 2014; Amooh and Bonsu, 2015). In particular, in semi-arid environments, soil evaporation accounts for about 30% loss of the total precipitation contributing significantly to the depletion of water in the soil profile (Wallace, 1991; Bonsu, 1997). Water absorbed moves quickly through sandy soils, but they retain very little in comparison with clay soil. Therefore, for an efficient water use, irrigation in sandy sites should be frequent and for short periods, to obtain a broader wetting area, providing more soil volume for roots to exploit, avoiding water moving beyond the root zone and contributing to soil leaching (Goldy, 2012).

Recently, an experimental site located in Lun soum (Tov province), has been used to evaluate the suitability of two tree species, *Populus sibirica* hort. ex Tausch and *Ulmus pumila* L. (Cho et al., 2019; Byambadorj et al., 2020). The *Populus* genus was selected for its high productivity, rapid re-sprouting capability, and easy and inexpensive propagation through cuttings (Mao et al., 2008; Stanturf and van Oosten, 2014; Jo and Park, 2017). The *Populus* genus has been used for almost 20 years in afforestation of Chinese steppes with appreciable results (Hu et al., 2008). The *Ulmus* genus has been chosen for its great adaptability to grow in soils with limited water and nutrient availability (Moore, 2003; Engelbrecht et al., 2005). For example, after 10 years of growth, the morpho-physiological analysis of both species highlighted that *P. sibirica* is characterized by higher productivity with respect to *U. pumila* as a consequence of its higher photosynthetic efficiency (Cho et al., 2019; Byambadorj et al., 2020). The lower productivity found in *U. pumila* trees has been related to a slower growth habit despite the higher water use efficiency than *P. sibirica* (Cho et al., 2019).

Management techniques such as watering regime and fertilization, are under investigation to test their efficiency to foster survival rate, growth, and long-term development of both tree species. Byambadorj et al. (2020) demonstrated that both survival rate and growth performance of *P. sibirica* trees depend strictly upon the applied management techniques whereas *U. pumila* trees are characterized by higher survival rates even without the application of irrigation. Moreover, increments observed in stem height and diameter could be positively correlated to watering regimes and negatively to fertilizers addition in the soil (Byambadorj et al., 2020).

Since soil characteristics may vary between types of soil and as results of watering and fertilization as well as the addition of amendments (Amendola et al., 2017; Montagnoli et al., 2021), the root developmental plasticity has been a major determinant for the success of land plants (Wilkinson, 2000; Hodge, 2004; Morris et al., 2017). The root system can be subdivided according to differences in diameter or tissue quality (Vogt et al., 1991; John et al., 2002; Zobel and Waisel, 2010; Montagnoli et al., 2018) of the various components, which correspond to different functions and carbon (C) input to the soil. In particular, the taproot represents the principal component of the root system (Esau, 1965) from which axis all lateral roots originate by the reiteration of a molecular and physiological branching mechanism (Chiatante et al., 2018). The taproot functions largely as tree anchorage, water transport, and storage of nutritional reserves (Noquet et al., 2001; Meuriot et al., 2003; Yang et al., 2014, 2016; Dumroese et al., 2019). Among all the lateral roots, the coarse fraction supports the development and function of the fine roots network (Di Iorio et al., 2011), transports water, and provides mechanical anchorage of the plant to its rooting environment. The finest fraction (diameter < 2 mm) of the root system represents the first plant-soil interface. It plays a crucial role in plant survival potential through associated mycorrhizae (Finér et al., 2011) that function in water and nutrient uptake and transport and exude carbohydrates that stimulate microbial activity (Coutts, 1983; Resh et al., 2003; Guo et al., 2013; Sun et al., 2017; Montagnoli et al., 2019a).

Roots regulate its architecture in response to signals in their local soil environment, such as water and nutrient availability, in addition to genetically determined developmental programmes (Chiatante et al., 2005; Montagnoli et al., 2018). Indeed, the continuous fluctuation in space and time of soil water and nutrients may induce the root system to respond through plastic morphological adaptation (Hodge, 2003; Hwang et al., 2007; Giehl et al., 2013) determining the volume of soil explored by a root system, and significantly impacting efficiency in acquiring resources (Morris et al., 2017). This might be particularly true for trees living in afforestation sites of the Mongolian steppe, which may use different strategies for their roots to adapt to variations in seasonal climates as well as different management techniques such as fertilization and watering (Ma et al., 2018).

We hypothesized that biomass partitioning of *P. sibirica* and *U. pumila* root systems would be affected by different management techniques and directly related to the enhancement of soil chemical characteristics. In particular, we would expect a higher fraction of fine roots associated with lower watering regimes and a higher fraction of coarse roots associated with higher watering regimes. To test our hypotheses, according to the watering regimes and fertilization type, soil chemical analysis was performed and root biomass was analyzed as a function of diameter classes. Our objective was to use a “shovelomics” approach, where a complete root system is excavated by hand, to understand how *P. sibirica* and *U. pumila* tree species modify their root system biomass partitioning and architecture through the complete hand-excavation of the roots system and its dissection into diameter classes.

## Materials and Methods

### Site Characteristics

The experimental site is located in Lun soum (Tuv province, Mongolia; 47°52'15.43″N, 105°10′46.4″E) on the right bank of the Tuul River, 135 km west of Ulaanbaatar at an elevation of 1,130 m a.s.l. The site extends for 2 ha within the forest nursery of the South Korea-Mongolia Joint *Green Belt* Plantation project in the Middle Khalkha dry steppe region (Ulziykhutag, 1989) that has been greatly degraded by intense livestock grazing.

The annual average temperature is 0.6 ± 0.45°C, and a summer average temperature is 16.29 ± 0.41°C. The mean air temperature of the warmest month (July) is 16°C, while that of the coldest month (January) is −22°C. The length of the growing season varies between 110 and 130 days. The average annual precipitation during the experiment (2000–2019) was 196 mm, according to the Lun soum weather station (NAMEM (The National Agency for Meteorology Environmental Monitoring of Mongolia), 2019). Precipitation usually occurs between June and August and accounts for 80–90% of the total annual rainfall. The mean annual potential evapotranspiration is 752 mm.

Vegetation is typical of the genuine dry bunchgrass steppe dominated by xerophytic and meso-xerophytic graminoids [e.g., *Stipa krylovii* Roshev. *, Cleistogenes squarrosa* (Trin.)*, Agropyron cristatum* (L.) Gaertn*, Artemisia frigida* (Willd.), and, in degraded lands *Artemisia adamsii* (Besser)*, Carex duriuscula* C.A. Mey. *, Leymus chinensis* (Trin.)] (Ulziykhutag, 1989; Lavrenko et al., 1991). Soil type is classified as Kastanozems (Loamic) type, more than 1 m and deep, immature, lacking horizontal development. The hardness of the topsoil is 4.5 kg cm^−2^, while that of the subsoil is 1.7 kg cm^−2^, as the topsoil is drier than the subsoil (IUSS Working Group WRB., 2015; Batkhishig, 2016). See Table 1 for chemical-physical details of the soil characteristics.

**Table 1:** Profile characteristics of the experimental site Kastanozems (Loamic) soil type.

									**Particle size distribution (%)**		
**Depth (cm)**	**pH**	**Carbon (%)**	**Organic matter (%)**	**Nitrate-Nitrogen (mg kg^**−1**^)**	**Electrical conductivity (dS m^**−1**^)**	**P_**2**_O_**5**_ (mg/100 g)**	**K_**2**_O**	**Rock content > 2 mm (%)**	**Sand (2–0.05 mm)**	**Silt (0.05–0.002 mm)**	**Clay (< 0.002 mm)**
0–10	7.77	0	0.892	6.03	0.060	1.02	10.2	0.42	68.6	21.9	9.5
10–20	7.33	0	0.806	7.89	0.024	0.81	7.8	1.21	71.8	19.0	9.2
20–30	7.20	0	0.813	7.40	0.026	1.06	6.7	0.76	71.5	19.2	9.3
30–40	7.17	0	0.880	7.82	0.025	0.68	5.5	1.55	72.1	18.7	9.2
40–50	7.26	0	0.842	6.41	0.025	1.77	5.5	1.36	72.5	17.4	10.1
50–60	7.18	0	0.801	6.69	0.028	0.77	5.5	1.02	71.9	17.6	10.5
60–70	7.23	0	0.824	8.17	0.033	1.60	5.5	1.08	71.8	18.3	9.9
70–80	7.17	0	0.734	6.69	0.035	1.48	5.5	1.19	71.8	18.4	9.8
80–90	7.30	0	0.512	7.50	0.074	1.27	5.5	1.26	76.2	13.2	10.6
90–100	8.04	0.95	0.248	7.47	0.073	1.10	5.5	0.18	77.7	12.1	10.2

*Data refer to two undisturbed plots outer the forest canopy for a total depth of 1 meter at 10 cm interval*.

### Plant Material and Management Techniques

In May 2011, 2-years-old seedlings of *Ulmus pumila* (grown from seeds) and *Populus sibirica* Tausch (obtained from 20 cm cuttings) were acclimated in the open Greenbelt project nursery and transplanted into holes 60 cm deep with a diameter of 50 cm. At transplanting time, *U. pumila* seedlings were 51 ± 1.14 cm in height with a diameter at root collar of 0.33 ± 0.01 cm, whereas *P. sibirica* seedlings were 68 ± 2.94 cm in height with a diameter at root collar of 0.51 ± 0.02 cm. Immediately after transplanting, a sufficient level of watering was supplied to individual trees by compensating non-leakage (CNL) button drippers.

**Figure 1:**
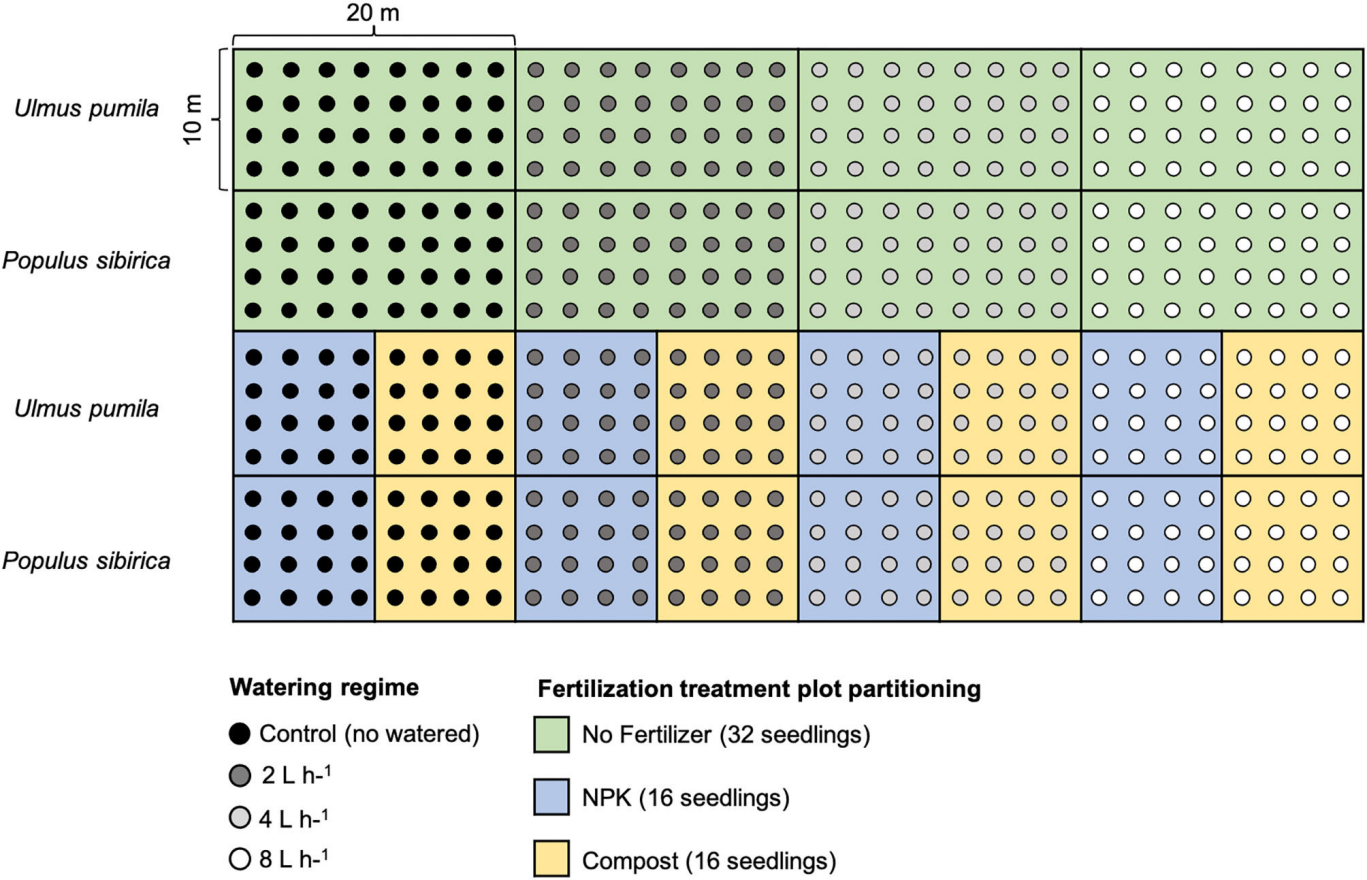
Planting scheme of the afforestation site. Green rectangular area (20 × 10 m) indicates the watering-only treatment (no fertilizers) with 32 seedlings for each of the watering regime. Light blue square areas (10 × 10 m) indicate the NPK fertilization with 16 seedlings for each of the watering regime. The yellow square areas (10 × 10 m) indicate Compost fertilization with 16 seedlings for each of the watering regime. Areas of the upper rows refer to *Ulmus pumila* tree plantation while areas of the lower rows refer to the *Populus sibirica* tree plantation. Black dots are relative to control trees (no watered), dark gray dots indicate 2 L h^−1^ watered trees, light gray dots indicate 4 L h^−1^ watered trees, and white dots indicate 8 L h^−1^ watered trees.

For each tree species, twelve plots were established in total: 4 plots characterized by the four watering regimes only with 32 seedlings per watering regime; 4 plots with different watering regimes and NPK addition with 16 seedlings per watering regime; 4 plots with different watering regimes and compost addition with 16 seedlings per watering regime. Seedlings were planted following rows distance of 2.5 m, and with a north-south orientation to ensure maximum light availability during the whole day (Johnson and Brandle, 2009) (Figure 1).

After seedling stabilization, water emitters were placed at a distance of 10 cm from the seedling, and four different watering regimes were applied: no watering (control), 2 L h^−1^ = 0.25 mm m^−2^, 4 L h^−1^ = 0.5 mm m^−2^, 8 L h^−1^ = 1 mm m^−2^. Seedlings were watered twice a week for the entire vegetative season (from the beginning of May to the end of August). The duration of each irrigation event was 5 h, provided through dripper buttons differing by the capacity of deliverable water. The irrigation hose system was connected to a water tank with a capacity of 50 m^3^. In each watering regime, two plots received a fertilizer treatment comprised of 500 g of solid granules of NPK or composted sheep manure (hereafter named compost). Fertilizers were mixed with natural soil before seedling transplantation.

### Soil Sampling Strategy and Chemical Analyses

To assess the effects of management techniques on soil chemical properties, at the beginning of July 2019 three soil samples were collected for each of the two tree species (*U. pumila* and *P. sibirica*), management techniques (four watering regimes alone and associated with NPK, and compost addition) and soil depth (0–20, 20–40, and 40–60 cm hereafter reported as mean 60 cm depth) at 20 cm distance from the stem (within the edges of the original planting hole), for a total of 216 samples. Soil samples were air-dried and passed through a 2 mm sieve. Soil organic matter (SOM) was measured by the K_2_Cr¬O_7_−H_2_SO_4_ oxidation method of Walkley and Black (Nelson and Sommers, 1996). Calcium carbon concentration was determined by the volumetric method (ASTM D4373-96, 1996). The pH was determined for a 1:2.5 air-dried soil/distilled water mixture using a glass electrode pH meter (Mongolian National Standard ISO 10390:^2001^, Mongolian National Standard ISO 10390:^2001^). The electrical conductivity (EC) was determined for a 1:5 air-dried soil/distilled water mixture using a platinum electrode. Available phosphorus (P_2_*O*_5_) was measured by molybdenum blue colorimetry, after (NH_4_)_2_CO_3_ digestion (Mongolian National Standard 3310:^1991^, Mongolian National Standard 3310:^1991^). Nitrate-nitrogen (NO_2_-N) was determined by using CH_3_COONa digestion and spectrocolorimetry. Potassium (K_2_O) was analyzed by flame spectrometry methods [SSIR (Soil Survey Investigations Report No.42), 2004].

### Biomass Measurement

In mid-July 2019, 10 years after seedling transplantation, 6 trees of each species and management technique were cut at the root collar and measured for height and DBH (see Byambadorj et al., 2020). For these 6 replicates for each thesis, we hand-excavated the root system to ~0.8–1 m in depth and to a distance of 1 m from the trunk. After cutting roots that were still attached to the soil, the root systems were carefully lifted and carried to an in-site field laboratory set up at the Lun soum nursery facility.

Roots were measured by a digital caliper at the branching point, detached and segregated by diameter classes according to the classification firstly suggested by Böhm (1979) and latterly revised by Zobel and Waisel (2010). In detail, all roots with diameters below 2 mm were selected as fine roots (FR). Roots with diameter above 2 mm were divided into 4 classes as follow: small (SR; 2 < d < 5 mm), medium (MR; 5 < d < 10 mm), large (LR; 10 < d < 20 mm), and very large (VLR; d > 20 mm). Moreover, the main root (hereafter termed taproot, TR), originating from seeds in the case of *U. pumila* and stem cutting in the case of *P. sibirica*, was also selected. To obtain biomass data, each root sample belonging to a specific diameter class was oven-dried at 105° C until constant weight and then weighed. In the case of root samples with a large volume, a 100 g fresh weight subsample was oven-dried and used to estimate the specific wood gravity to be applied to the whole sample.

### Statistical Analysis

Soil statistical analysis was computed using the SAS software package, version 9.4 (SAS Institute Inc., Cary, North Carolina, USA). One-way analysis of variance (ANOVA) with Duncan's multiple range test (DMRT) was used for multiple comparisons of soil chemical properties among treatments and species. Differences were considered significant at *p* < 0.05.

For root biomass, a two-way ANOVA for each root class was carried out to test the effect of nutrient and water regime treatments. When needed, the dependent variables were square-root or log-transformed to ensure normal distributions and equal variances. Omega squared values (ω^2^) for each predictor were calculated in order to show the variation in the dependent variable attributable to the independent variable (predictors). *Post-hoc* LSD-tests were conducted to detect overall differences between watering regimes and type of nutrient treatments. For the ratio between each root class and total root biomass, a categorical principal component analysis (CATPCA) was performed to represent the variability of our dataset to a nominal (nutrient treatment) and a numerical (water regime) independent variable. Moreover, to quantify how the root system growth was influenced by both independent variables, a linear regression model was calculated by relating total root biomass with water regime for each nutrient treatment and species. Differences were considered significant at *p* < 0.05. Statistical analysis was carried out with SPSS 17.0 (SPSS Inc., Chicago IL, USA).

## Results

### Soil Chemical Properties

Soil pH under the *Ulmus pumila* was not influenced by the different watering regimes (Table 2). Values of pH for the soil amended with NPK showed a significant decrease of 12.5% only in non-watered soil (control). The same pattern was found in the Compost addition (Table 2). A slight but insignificant decrease was found in non-watered soil while no differences were detected when different watering regimes were added (Table 2). Soil organic matter (%) did not significantly change in watering-only soil when different watering regimes were considered. No watered soil treated with NPK addition showed a slight significant increase of soil organic matter (Table 2). When watering regimes were added to the NPK, organic matter values decreased to levels measured for watering-only soil. The highest values of organic matter were measured in Compost treated soil with no watering (control) (Table 2). When watering regimes were added to the Compost treated soil, organic matter significantly decreased to the same values measured for watering-only soil. Nitrate-Nitrogen concentration in the soil was not influenced by the watering-only regimes. When both NPK and Compost were added, soil Nitrate-Nitrogen concentration significantly increased with respect to the watering-only soil (around 177% for both treatments; Table 2). Also, in this case the addition of watering regimes did not significantly change the Nitrate-Nitrogen soil concentration. Both phosphorus (P_2_O_5_) and potassium (K_2_O) did not significantly change if watering regimes and fertilizers were considered (Table 2).

**Table 2:** Soil chemical properties for different management techniques and watering regimes for *Ulmus pumila* and *Populus sibirica*.

**Plant species**	**Management technique**	**Watering regimes**	**pH**	**Organic matter (%)**	**Nitrate-Nitrogen (mg kg^**−1**^)**	**P_**2**_O_**5**_ (mg/100 g)**	**K_**2**_O**
*U. pumila*	Watering only	Control	7.83 ± 0.10^a^	0.82 ± 0.05^def^	3.1 ± 0.4^d^	0.84 ± 0.12^b^	4.7 ± 1.6^b^
		2 L h^−1^	7.68 ± 0.05^a^	0.94 ± 0.03^bcde^	3.5 ± 0.3^d^	0.60 ± 0.84^b^	5.5 ± 1.8^ab^
		4 L h^−1^	7.55 ± 0.07^a^	0.90 ± 0.02^cde^	2.0 ± 0.4^d^	2.80 ± 1.19^a^	6.7 ± 1.8^ab^
		8 L h^−1^	7.43 ± 0.02^a^	0.96 ± 0.06^bcd^	2.7 ± 0.4^d^	0.78 ± 0.43^b^	6.3 ± 2.1^ab^
	Watering and NPK	Control	6.85 ± 0.03^b^	1.12 ± 0.12^b^	7.8 ± 0.4^abc^	0.78 ± 0.18^b^	9.0 ± 1.2^a^
		2 L h^−1^	7.87 ± 0.19^a^	0.75 ± 0.09^f^	7.1 ± 0.4^bc^	0.63 ± 0.41^b^	8.2 ± 2.7^ab^
		4 L h^−1^	7.84 ± 0.14^a^	0.90 ± 0.03^cde^	6.8 ± 0.7^bc^	1.00 ± 0.14^ab^	6.6 ± 0.7^ab^
		8 L h^−1^	7.76 ± 0.14^a^	0.89 ± 0.06^cdef^	7.1 ± 0.2^bc^	0.65 ± 0.30^b^	5.5 ± 1.2^ab^
	Watering and compost	Control	7.35 ± 0.42^ab^	1.50 ± 0.10^a^	7.7 ± 0.4^abc^	1.31 ± 0.65^ab^	9.0 ± 1.2^a^
		2 L h^−1^	7.78 ± 0.12^a^	1.04 ± 0.03^bc^	8.1 ± 0.3^ab^	1.24 ± 0.41^ab^	7.1 ± 1.0^ab^
		4 L h^−1^	7.85 ± 0.29^a^	0.78 ± 0.04^ef^	6.2 ± 0.7^c^	1.08 ± 0.35^ab^	6.3 ± 1.4^ab^
		8 L h^−1^	7.88 ± 0.13^a^	0.82 ± 0.04^def^	9.4 ± 1.8^a^	0.63 ± 0.20^b^	5.5 ± 1.2^ab^
*P. sibirica*	Watering only	Control	7.75 ± 0.02^a^	0.90 ± 0.02^ab^	5.2 ± 0.6^e^	0.43 ± 0.17^c^	7.5 ± 1.9^a^
		2 L h^−1^	7.30 ± 0.13^bc^	1.00 ± 0.11^a^	5.5 ± 0.1^e^	0.77 ± 0.09^abc^	6.7 ± 1.2^a^
		4 L h^−1^	7.15 ± 0.04^cd^	0.84 ± 0.08^abc^	9.6 ± 0.1^bcd^	0.82 ± 0.04^abc^	7.8 ± 2.3^a^
		8 L h^−1^	6.79 ± 0.06^d^	0.89 ± 0.00^ab^	6.0 ± 0.3^e^	0.85 ± 0.27^abc^	5.9 ± 1.0^a^
	Watering NPK	Control	7.65 ± 0.25^ab^	0.77 ± 0.07^bc^	7.9 ± 0.4^d^	0.58 ± 0.21^bc^	7.5 ± 1.9^a^
		2 L h^−1^	7.47 ± 0.10^abc^	0.88 ± 0.08^ab^	7.9 ± 0.5^d^	0.40 ± 0.11^c^	6.3 ± 0.8^a^
		4 L h^−1^	7.63 ± 0.18^ab^	0.69 ± 0.02^c^	10.3 ± 0.4^abc^	0.50 ± 0.09^c^	7.5 ± 2.6^a^
		8 L h^−1^	7.21 ± 0.16^c^	0.86 ± 0.07^ab^	11.4 ± 0.1^a^	0.60 ± 0.07^abc^	9.8 ± 2.6^a^
	Watering compost	Control	7.29 ± 0.18^bc^	0.77 ± 0.08^bc^	10.4 ± 1.3^abc^	0.70 ± 0.12^abc^	6.3 ± 0.8^a^
		2 L h^−1^	7.45 ± 0.02^abc^	0.77 ± 0.01^bc^	9.6 ± 0.8^cd^	1.09 ± 0.22^ab^	7.1 ± 1.6^a^
		4 L h^−1^	7.32 ± 0.01^bc^	0.95 ± 0.04^a^	11.2 ± 0.4^abc^	0.47 ± 0.16^c^	7.1 ± 1.6^a^
		8 L h^−1^	7.75 ± 0.19^a^	0.98 ± 0.04^a^	11.3 ± 0.9^abc^	1.14 ± 0.33^a^	9.0 ± 3.5^a^

*Values represent the mean of 3 soil depth (0–20, 20–40, and 40–60 cm) and 3 samples (n = 3) ± SE. Letters indicate significant differences among factors for each species*.

Soil pH under *P. sibirica* watering-only treatment progressively decreased with increasing level of watering regimes (Table 2). When NPK was added soil pH for both control and 2L h^−1^ was similar to the watering-only soil and was significantly higher when 4 and 8 L h^−1^ watering regimes were applied (6.7 and 6.2%, respectively; Table 2). When Compost was added to the soil, pH values were significantly higher when 8 L h^−1^ watering regime was applied (106% in respect to the control value). Soil organic matter for watering-only soils did not differ among the different watering regimes (Table 2). Similarly, in the case of soil treated with NPK, organic matter did not differ from the watering-only soils and did not show significant variation among different watering regimes with the only exception of the of 4 L h^−1^ watering regime. In the case of soil treated with Compost, organic matter was significantly higher in the 4 and 8 L h^−1^ among watering regimes (around 25% for both treatments; Table 2). Nitrate-Nitrogen did not significantly change with watering regimes in the watering-only soil with the exception of the of 4 L h^−1^ watering regime. When both NPK and Compost treated soil were analyzed, Nitrate-Nitrogen concentration was higher than watering-only soils (42.6 and 61.6%, respectively; Table 2). Higher values among watering regimes were found in NPK treated-soils when 4 and 8 L h^−1^ of water were added. When Compost was added to the soil, no differences were found among watering regimes. Both phosphorus (P_2_O_5_) and potassium (K_2_O) did not significantly change if watering regimes and fertilizers were considered (Table 2).

### Plant Survival Rate

Six months after transplanting in the field (September 2011), the survival rate of seedlings was 100% for both *U. pumila* and *P. sibirica*. Overall survival rate of all treatments 8 years later (2019) was 92% for *U. pumila* and 82% for *P. sibirica*. In particular, the number of surviving *P. sibirica* trees was reduced to 41, 13, and 37%, respectively for control (no watering), control plus NPK, and control plus compost management techniques. In the case of *U. pumila* trees survival rate was reduced to 62% only for control plus NPK management technique.

### Root Biomass Partitioning

#### Fine Roots (FR)

**Figure 2:**
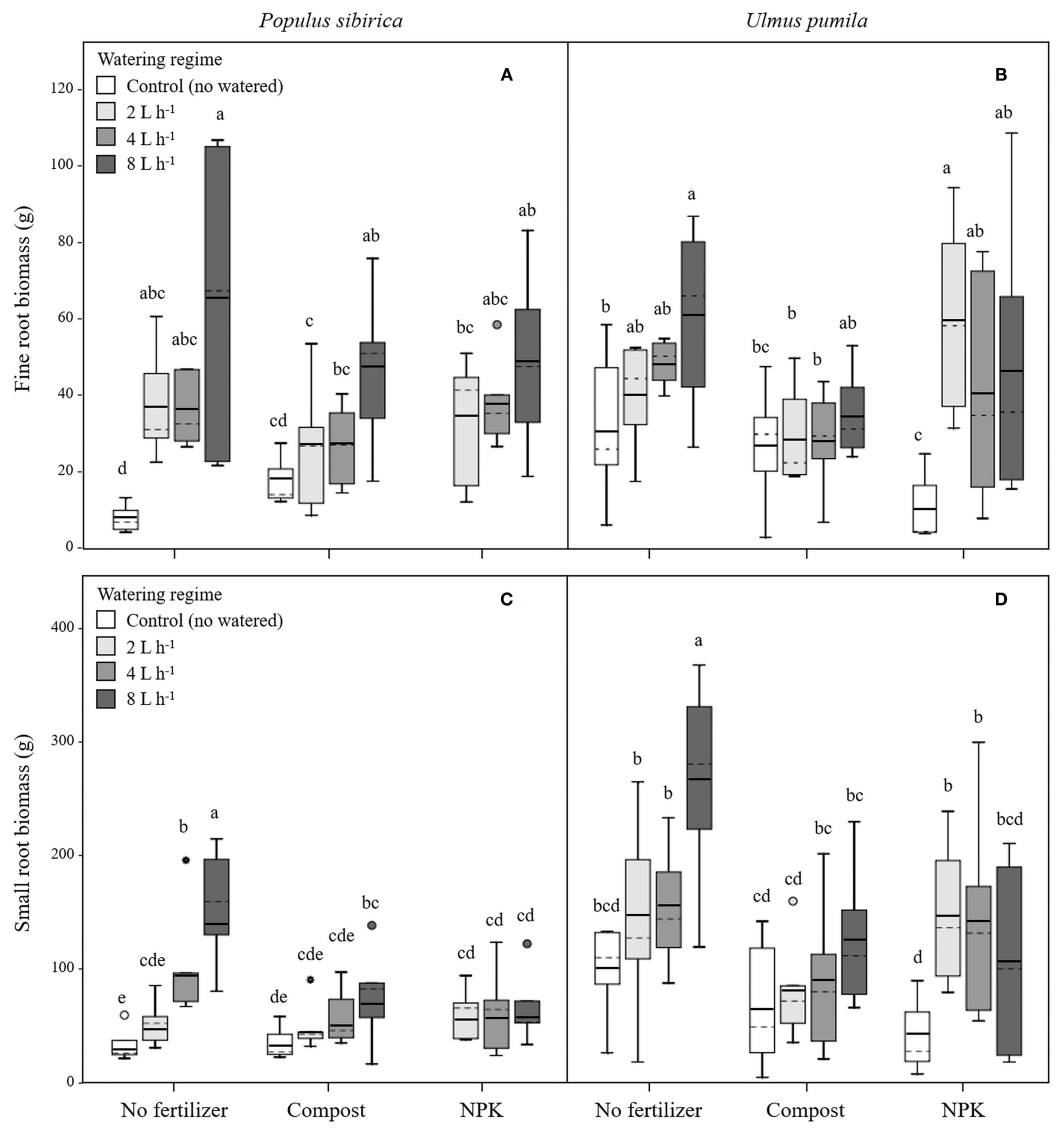
Fine and small root biomass for *P. sibirica* **(A,C)** and *U. pumila* **(B,D)** for different watering regimes and by different fertilization treatments. Different letters indicate significant differences (*p* < 0.05) among watering regimes within each fertilization treatment. Vertical boxes represent ~50% of the observations and lines extending from each box are the upper and lower 25% of the distribution. Within each box, the solid horizontal line is the mean value and the dotted line is the median.

In *P. sibirica* trees the different watering regimes significantly influenced the FR biomass (*p* < 0.001) accounting for 28.3% of the data variation (Table 3). The FR biomass measured in trees supported by different watering regimes (2, 4, and 8 L h^−1^) was significantly higher than in control trees (5, 4, and almost 9-fold, respectively; Figure 2A). FR biomass did not differ between the three watering regimes despite that trees grown with the 8 L h^−1^ watering regime presented the highest values. On the contrary, ANOVA analysis did not reveal a significant influence of the fertilization treatment (*p* = 0.733) (Table 3). *P. sibirica* trees treated with the addition of compost showed the same pattern with the highest and lowest values in the case of 8 L h^−1^ and no watering regimes, respectively. No differences in FR were detected among control and 2 and 4 L h^−1^ treated trees. When soil was fertilized with NPK, the highest values of FR biomass were found with trees treated to 8 L h^−1^ watering regime, despite that the differences with values found in trees treated with 4 and 2 L h^−1^ watering regimes were not significant. Data on control trees are missing due to mortality of all trees. Although not significantly different, slightly higher values of FR were detected in 8 L h^−1^ trees subject to watering only in comparison with trees subject to watering and NPK addition (Figure 2A).

**Table 3:** Two-way ANOVA results (predictors: WAT, water regime; FER, fertilization treatments) for biomass of each root diameter class.

**Plant species**	**Root class (biomass)**	**Predictor**	***F***	***p***	****ω**^2^**
*P. sibirica*	Fine root	WAT	10.26	**<** **0.001**	0.283
		FER	0.31	0.733	0
	Small root	WAT	8.80	**<** **0.001**	0.259
		FER	4.95	**0.010**	0.087
	Medium root	WAT	3.32	**0.027**	0.096
		FER	0.58	0.561	0
	Large root	WAT	0.46	0.715	0
		FER	0.67	0.515	0
	Very large	WAT	7.23	**0.001**	0.264
		FER	0.78	0.467	0
	Tap root	WAT	14.97	**<** **0.001**	0.398
		FER	2.54	0.089	0.029
	Total root	WAT	14.75	**<** **0.001**	0.395
		FER	0.65	0.525	0
*U. pumila*	Fine root	WAT	6.00	**0.001**	0.158
		FER	3.39	**0.041**	0.050
	Small root	WAT	5.81	**0.002**	0.142
		FER	7.48	**0.001**	0.127
	Medium root	WAT	0.06	0.980	0
		FER	1.58	0.214	0.017
	Large root	WAT	6.67	**0.001**	0.165
		FER	6.36	**0.003**	0.104
	Very large	WAT	2.57	0.062	0.058
		FER	0.70	0.502	0
	Tap root	WAT	6.26	**0.001**	0.171
		FER	1.30	0.281	0.006
	Total root	WAT	2.99	**0.001**	0.155
		FER	1.90	0.058	0.041

*F-value (F), p-value (p), and omega square value are shown. Bold values indicate statistical significance (p < 0.05)*.

In *U. pumila* trees the different watering regimes significantly influenced the FR biomass (*p* = 0.001) accounting for 15.8% of the data variation (Table 3). The FR biomass measured in trees supported with different watering regimes only, were the highest and the lowest in 8 L h^−1^ and control trees, respectively, while intermediate values were measured for trees subject to the addition of 2 and 4 L h^−1^, which were similar each other (Figure 2B). Fertilization treatment significantly influenced the FR biomass (*p* = 0.041) accounting for 5% of the data variation (Table 3). When the soil was fertilized with compost, FR values were similar across all watering regimes and of lowest magnitude in comparison with trees subject to watering only. When NPK was added to the soil the lowest value of FR was measured across all watering regimes and management treatments considered. FR values measured for trees with 4, and 8 L h^−1^ were similar each other and compared to the other management techniques while FR values for the 2 L h^−1^ where the highest (Figure 2B).

#### Small Roots (SR)

In *P. sibirica* trees the different watering regimes significantly influenced the SR biomass (*p* < 0.001) accounting for 25.9% of the data variation (Table 3). The biomass of SR measured in trees supported by the 8 L h^−1^ watering regime was the highest measured across all regimes (Figure 2C). Trees supported by the 4 L h^−1^ regime had intermediate values of SR. The SR biomass for trees supported by the addition of 2 L h^−1^ watering regime did not differ from the control trees, which showed the lowest biomass values (Figure 2C). Fertilization treatment significantly influenced the SR biomass (*p* = 0.010) accounting for 8.7% of the data variation (Table 3). Trees supported by the addition of compost showed biomass values 2.2-fold significantly higher than unwatered trees only in the case of 8 L h^−1^. Moreover, SR biomass of the trees treated with 4 and 8 L h^−1^ plus compost was significantly lower (−36.3 and −50.9%, respectively) than that of trees treated alone with the same watering regime; the same pattern was observed for trees supported by the addition of NPK (−28.3 and −59.2%, respectively). For this treatment condition, unwatered trees had a 100% mortality and thus were not detectable, while trees watered at the three levels showed the same values of lower magnitude when compared with watered trees alone (Figure 2C).

In *U. pumila* trees the different watering regimes significantly influenced the SR biomass (*p* = 0.002) accounting for 14.2% of the data variation (Table 3). Values of SR biomass measured in trees under different watering regimes were the highest and the lowest in 8 L h^−1^ and control trees, respectively, while trees subject to the addition of 2 and 4 L h^−1^ had intermediate values (Figure 2D). Fertilization treatment significantly influenced the SR biomass (*p* = 0.001) accounting for 12.7% of the data variation (Table 3). When compost was applied, SR biomass was not significantly different from irrigation alone (Figure 2D). SR biomass in the compost plus watering of 8 and 2 L h^−1^ treatments was significantly lower than values measured for trees with the same watering regimes only, while no differences were detected for control and 4 L h^−1^. When NPK was applied, SR biomass was higher in trees watered at 2 and 4 L h^−1^ in comparison with unwatered trees (Figure 2D) while the 8 L h^−1^ trees did not differ from unwatered trees. Finally, values of SR biomass in trees treated with NPK were similar to those measured for compost trees across all watering regimes, and trees supported with 8 L h^−1^ had less SR biomass than trees with watering regimes only (Figure 2D).

#### Medium Roots (MR)

**Figure 3:**
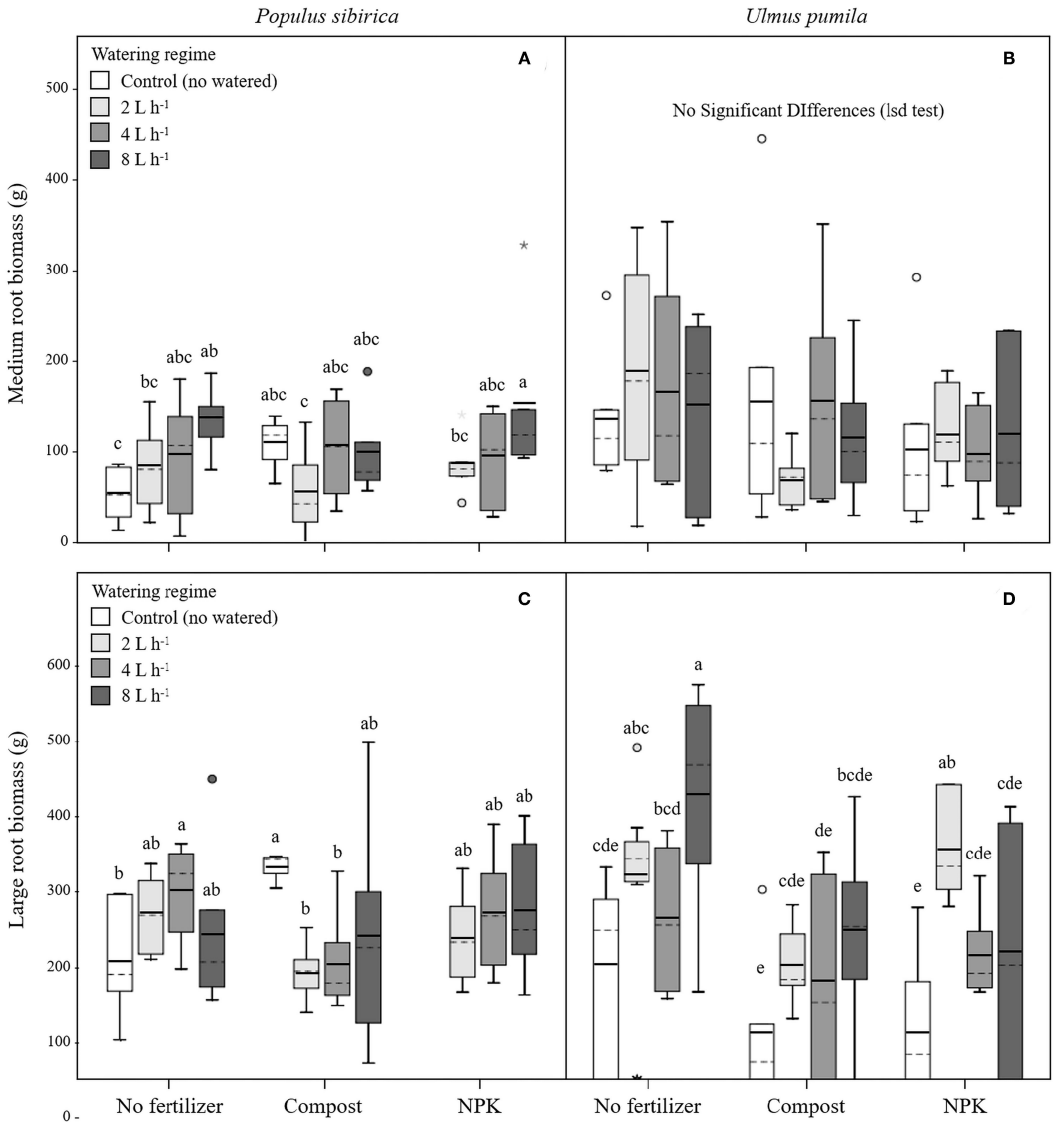
Medium and large root biomass for *P. sibirica* **(A,C)** and *U. pumila* **(B,D)** for different watering regimes and by different fertilization treatments. Different letters indicate significant differences (*p* < 0.05) among watering regimes within each fertilization treatment; absence of letters reflects that no significant difference was detected. Vertical boxes represent ~50% of the observations and lines extending from each box are the upper and lower 25% of the distribution. Within each box, the solid horizontal line is the mean value and the dotted line is the median.

In *P. sibirica* trees the different watering regimes significantly influenced the MR biomass (*p* = 0.027) accounting for 9.6% of the data variation (Table 3). MR biomass was similar across the three levels of watering (2, 4, and 8 L h^−1^), and a significant difference was detected only between no watered plants (control) and the 8 L h^−1^ watering regime, which was 2.5-fold higher (Figure 3A). ANOVA analysis did not reveal a significant influence of the fertilization treatment (*p* = 0.561) on the MR biomass (Table 3). Trees fertilized with compost had similar MR biomass values across all watering regimes. Furthermore, these values did not differ from MR biomass of irrigated trees. Similarly, trees fertilized with NPK showed the highest and the lowest values of MR biomass at 2 and 8 L h^−1^ respectively, with intermediate values for the 4 L h^−1^ trees (Figure 3A). Also, MR biomass of trees fertilized and watered did not differ from for trees irrigated the same watering regime alone. In the case of *U. pumila* trees ANOVA analysis did not reveal a significant influence of either watering (*p* = 0.980) or fertilization (*p* = 0.214) treatments (Table 3). Indeed, trees did not show any difference in MR biomass among all watering regimes and fertilizers used (Figure 3B).

#### Large Roots (LR)

In *P. sibirica* trees the ANOVA analysis did not reveal a significant influence on the LR biomass of both watering (*p* = 0.715) and fertilization (*p* = 0.515) treatments (Table 3). LR biomass in *P. sibirica* trees differed only for the 4 L h^−1^ trees (Figure 3C). When trees were additionally treated with compost, the highest and the lowest values of LR biomass were measured, respectively for the unwatered, 2 and 4 L h^−1^ trees. The trees watered at 8 L h^−1^ had intermediate values (Figure 3C). Trees fertilized with NPK did not show any difference across the three levels of watering regimes, and these values did not differ from trees irrigated alone (Figure 3C).

In *U. pumila* trees the different watering regimes significantly influenced the LR biomass (*p* = 0.001) accounting for 16.5% of the data variation (Table 3). LR biomass of *U. pumila* trees supported by 8 L h^−1^ was 2.5-fold significantly higher than biomass of unwatered trees (Figure 3D). LR biomass for 2 L h^−1^ trees was intermediate, being similar to the 8 and 4 L h^−1^ and to the no watered trees. Fertilization treatment significantly influenced the LR biomass (*p* = 0.003) accounting for 10.4% of the data variation (Table 3). When compost was added, no differences were detected across all irrigation treatments. LR biomass for trees treated with compost plus irrigation was similar to trees with irrigation alone; the only exception was the 8 L h^−1^ trees, which were 47.8% significantly lower (Figure 3D). When NPK was added, trees supported by 2 L h^−1^ had the highest LR biomass, which was similar to the trees supported by the 8 L h^−1^ watering-only regime.

#### Very Large Roots (VLR)

**Figure 4:**
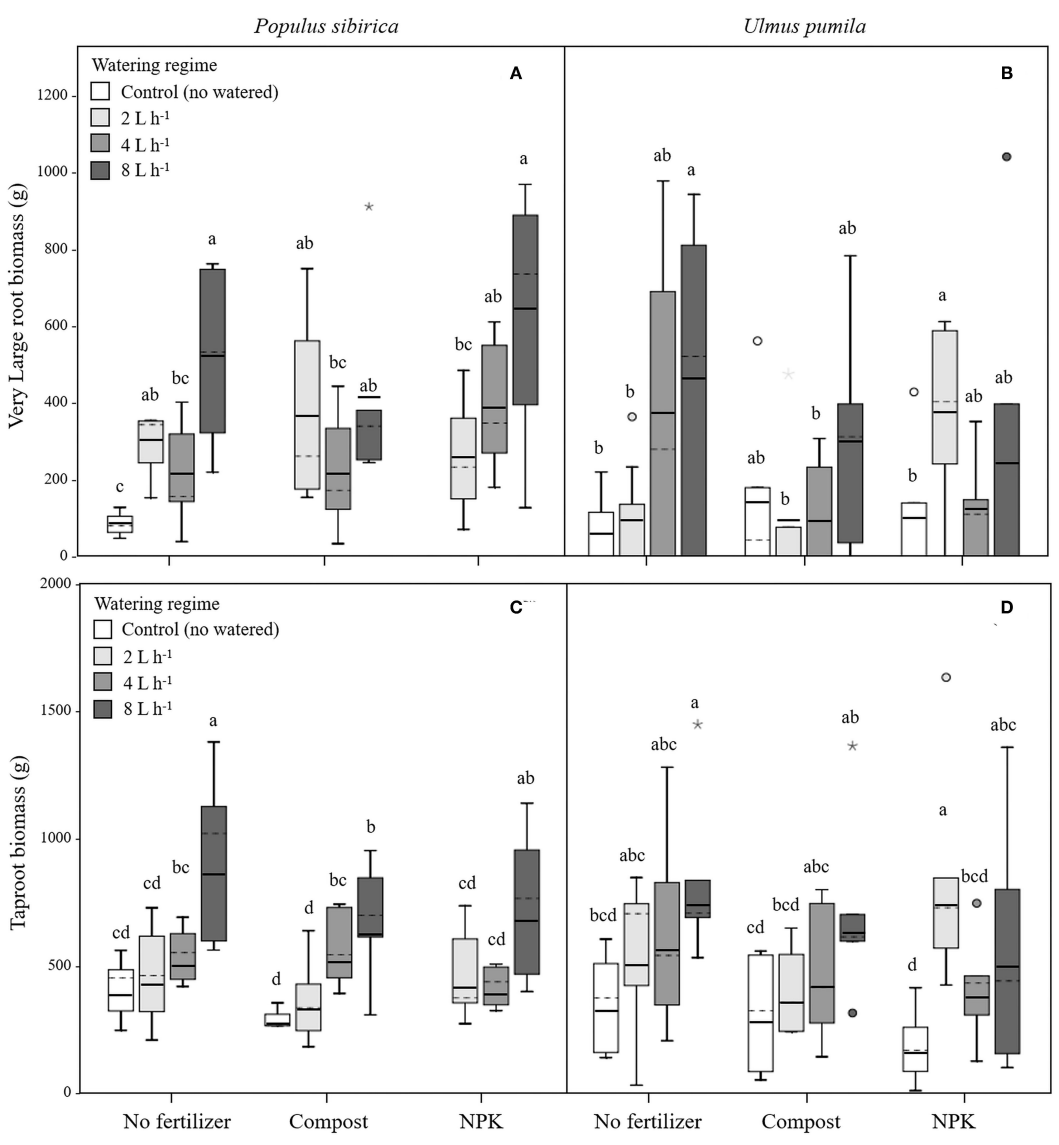
Very large and tap- root biomass for *P. sibirica* **(A,C)** and *U. pumila* **(B,D)** for different watering regimes and by different fertilization treatments. Different letters indicate significant differences (*p* < 0.05) among watering regimes within each fertilization treatment. Vertical boxes represent ~50% of the observations and lines extending from each box are the upper and lower 25% of the distribution. Within each box, the solid horizontal line is the mean value and the dotted line is the median.

In *P. sibirica* trees the different watering regimes significantly influenced the VLR biomass (*p* = 0.001) accounting for 26.4% of the data variation (Table 3). Biomass of VLR of trees supported by the 2 and 8 L h^−1^ watering regime was significantly higher, 3.6 and 9.3-fold, respectively, than unwatered trees, which showed the lowest values (Figure 4A). ANOVA analysis did not reveal a significant influence of the fertilization treatment (*p* = 0.467) (Table 3). Compost additions had no effect on VLR biomass across the three levels of watering. Both 2 and 8 L h^−1^ plus compost were the same as the highest value of biomass measured for the trees watered at 8 L h^−1^ alone (Figure 4A). Addition of NPK showed the highest and the lowest values of VLR biomass for 8 and 2 L h^−1^, respectively; 4 L h^−1^ had intermediate values (Figure 4A).

In the case of *U. pumila* trees ANOVA analysis did not reveal a significant influence of either watering (*p* = 0.062) or fertilization (*p* = 0.502) treatments (Table 3). VLR biomass values were significantly higher in trees treated by 8 L h^−1^ than unwatered and 2 L h^−1^ trees; trees in the 4 L h^−1^ treatment had intermediate values (Figure 4B). When compost was added VLR biomass values did not differ from those measured for trees supported by watering alone. The NPK treatment produced the highest and the lowest VLR biomass values for the 2 L h^−1^ and unwatered trees. Trees treated with 4 and 8 L h^−1^ had intermediate values. VLR values were similar to those measured for trees supported with watering regime only (Figure 4B).

#### Tap Root (TR)

In *P. sibirica* trees the different watering regimes significantly influenced the TR biomass (*p* < 0.001) accounting for 39.8% of the data variation (Table 3). TR biomass measured in *P. sibirica* trees treated with 8 L h^−1^ was significantly higher (1.7 and 2.2-fold, compared to 4 and 2 L h^−1^) than in trees in the other watering levels including the unwatered (Figure 4C). ANOVA analysis did not reveal a significant influence of the fertilization treatment (*p* = 0.089) on the TR biomass (Table 3). Compost addition had the highest values of TR biomass in both the 4 and 8 L h^−1^ treatments and the lowest for 2 L h^−1^ and unwatered trees (Figure 4C). TR biomass of 8 L h^−1^ trees with compost addition was 27.4% significantly lower than trees at the same level of watering alone. Similarly, *P. sibirica* trees treated with added NPK had the highest TR biomass values for trees supported with 8 L h^−1^ and the lowest for the 2 and 4 L h^−1^ treatments (Figure 4C). The highest TR values detected for 8 L h^−1^ trees were similar to the values measured for trees supported with same watering level only.

In *U. pumila* trees the different watering regimes significantly influenced the TR biomass (*p* = 0.001) accounting for 17.1% of the data variation (Table 3). *U. pumila* trees had the highest values of TR biomass when trees were watered with 8 L h^−1^ (Figure 4D). TR biomass gradually declined with lower levels of watering, with the lowest values for unwatered trees. Fertilization treatment did not influence the TR biomass (*p* = 0.281) (Table 3). A similar pattern was observed when compost was added although TR biomass for the three watering levels did not differ from each other (Figure 4D). TR biomass did not differ across the watering regimes with NPK addition with the only exception the trees supported with 2 L h^−1^, which had the highest mean biomass value.

#### Total Root Biomass (TRB)

**Figure 5:**
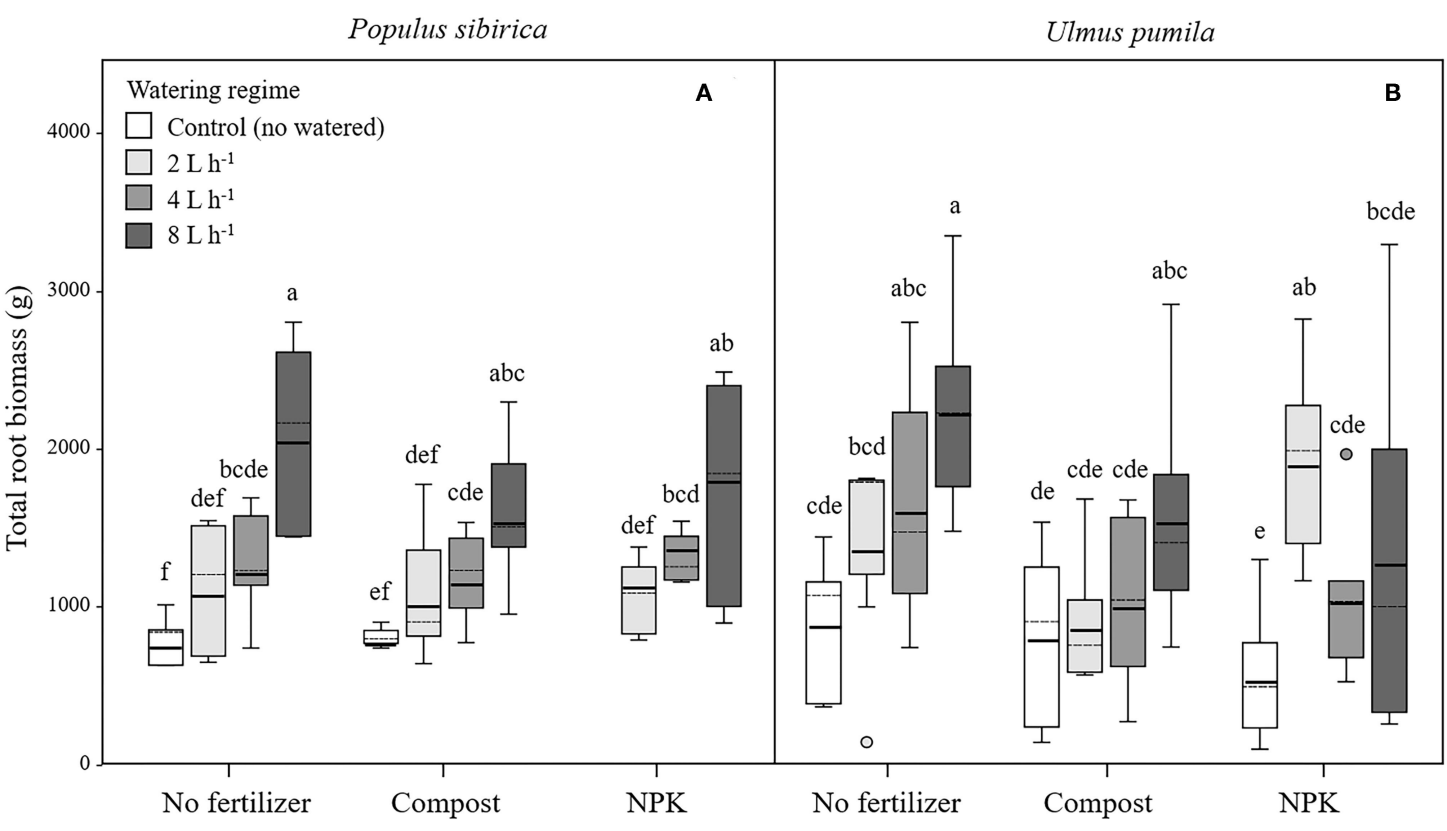
Total root biomass for *P. sibirica* **(A)** and *U. pumila* **(B)** for different watering regimes and by different fertilization treatments. Different letters indicate significant differences (*p* < 0.05) among watering regimes within each fertilization treatment. Vertical boxes represent ~50% of the observations and lines extending from each box are the upper and lower 25% of the distribution. Within each box, the solid horizontal line is the mean value and the dotted line is the median.

In *P. sibirica* trees the different watering regimes significantly influenced the TRB (*p* < 0.001) accounting for 39.5% of the data variation (Table 3). *P. sibirica* had the highest TRB values for trees supported with 8 L h^−1^ and the lowest in the unwatered trees (Figure 5A). Trees in the 2 and 4 L h^−1^ watering regimes showed intermediate values. Similar patterns were observed when compost and NPK was added, although 8 and 4 L h^−1^ did not differ from each other. Indeed, ANOVA analysis did not reveal a significant influence of the fertilization treatment (*p* = 0.525) (Table 3).

TRB biomass of *U. pumila* trees followed the same trend as was observed for *P. sibirica* with the highest and the lowest values measured for trees at the highest watering level and the unwatered trees (Figure 5B). For this tree species watering treatment significantly influenced the TRB (*p* = 0.001) while fertilization treatment had no influence on the data variation (*p* = 0.058).

#### Categorical Principal Component Analysis (CATPCA) of Biomass Data

**Figure 6:**
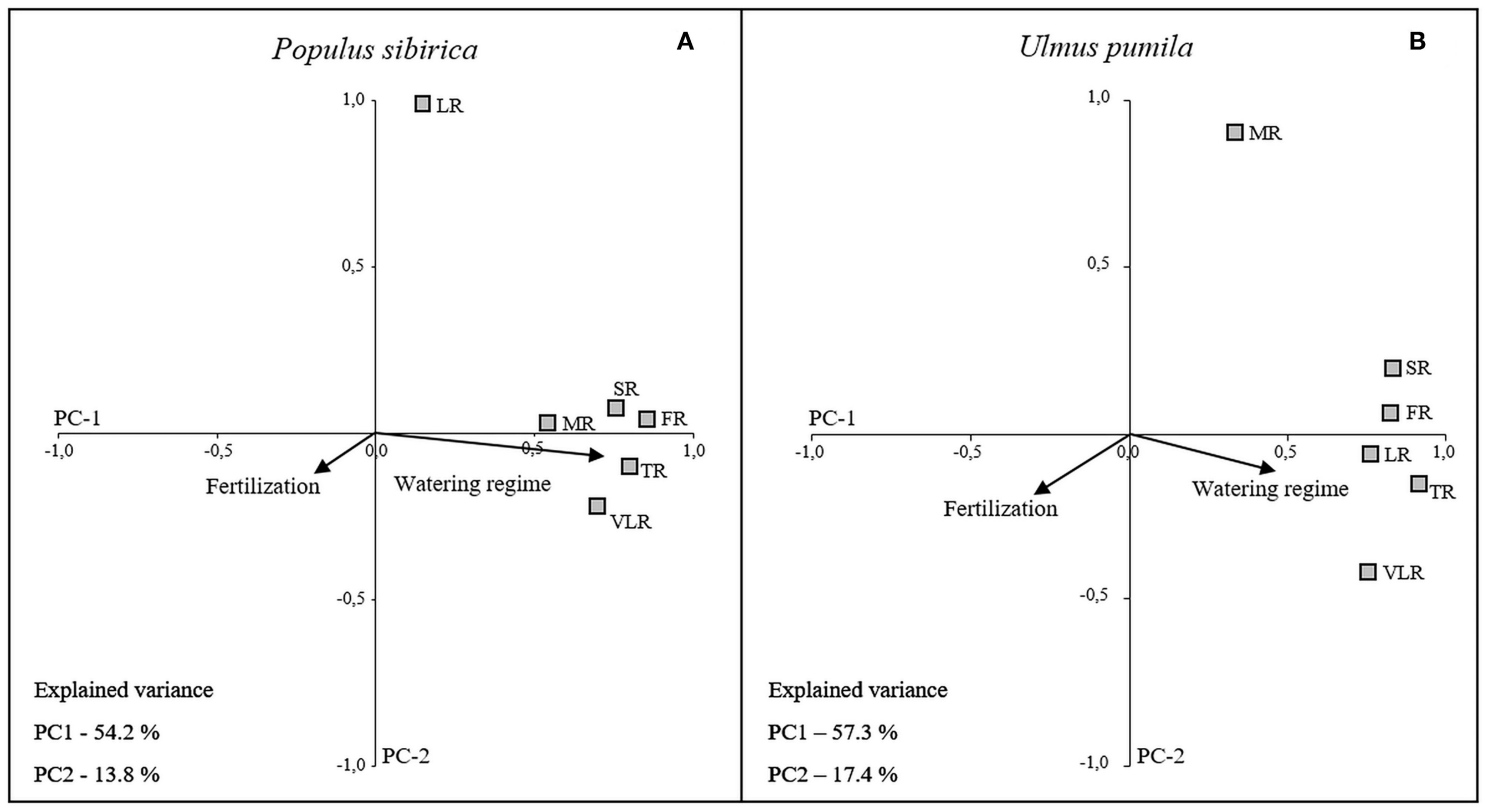
Categorical Principal Component Analysis (CATPCA) biplot of *P. sibirica* **(A)** and *U. pumila* **(B)** indicating the different root classes biomass projection in relation with watering regime and fertilization. Tightly clustered variables are positively related, while variables at different ends of the figure are negatively related. Arrows represents the mean influence magnitude for each factor.

The first two principal components (PC1 and PC2) accounted for 68% (*P. sibirica:* PC1 54.2%, PC2 13.8%) and 74.7% (*U. pumila:* PC1 57.3%, PC2 17.4%) of the data variance. The loading plots of each root diameter class (Figure 6) was distributed along the PC1 axis with positive correlation to the water regime (higher the irrigation, higher the root mass) for both tree species, although *U. pumila* (Figure 6B) was influenced with lower magnitude than *P. sibirica* (Figure 6A). The only exception was represented by LR for *P. sibirica* and MR for *U. pumila* that were better explained by the PC2 axis showing no correlation with water regime. Moreover, the analysis of PC1 coordinate showed that fertilization treatments, being a categorical variable, had a significant influence on root biomass, although of less intensity than water regime (Figure 6). Root biomass of *U. pumila* was influenced by the fertilizers with higher magnitude than *P. sibirica*.

#### Regression Biomass Growth Model

**Figure 7:**
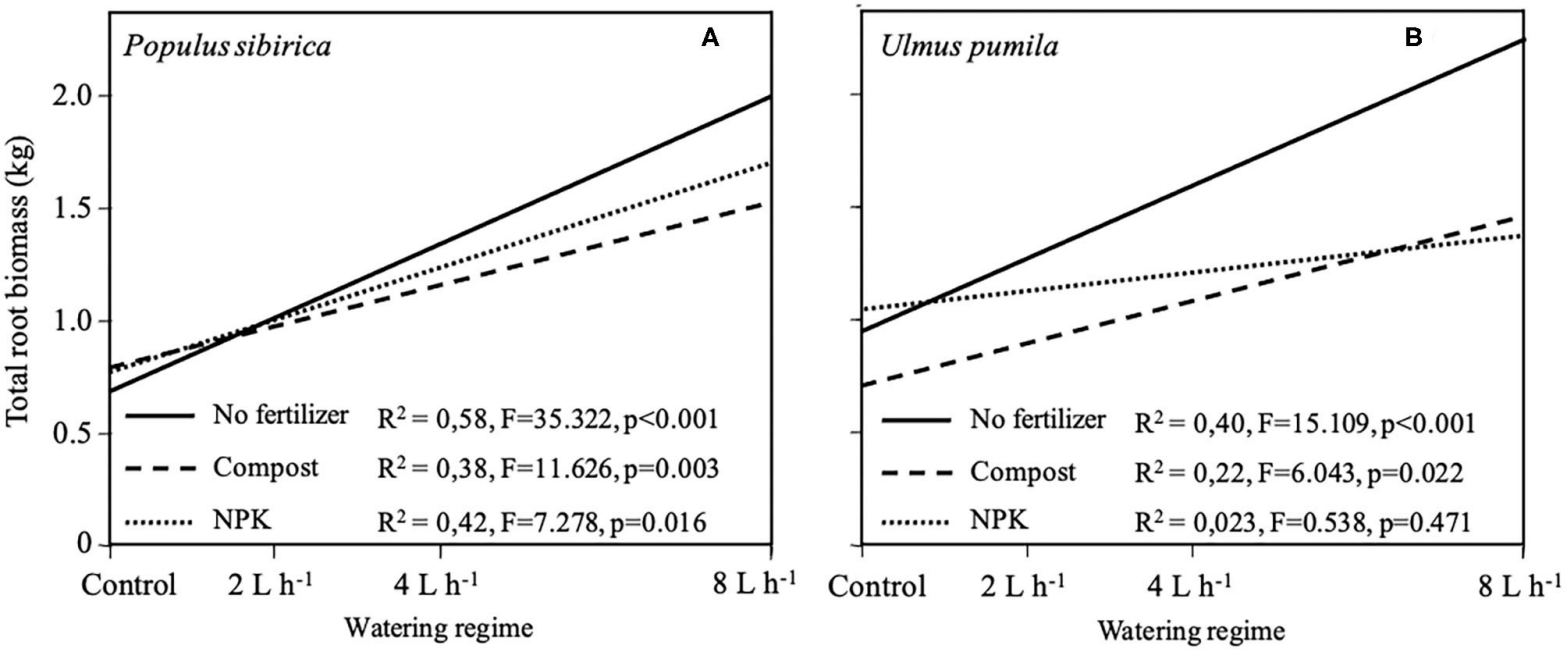
Regression models of total root biomass for *P. sibirica* **(A)** and *U. pumila* **(B)** calculated by destructive sampling in relation with different watering regimes and fertilization treatment (No fertilizers, solid line; Compost, dashed line; NPK, dotted line).

Regression growth models were obtained from the relationship of the total root system biomass (TRB) with different watering regimes for each of the fertilizer types used and the two tree species analyzed (Figure 7). In the case of *P. sibirica*, TRB significantly increased (*p* < 0.001; F = 35.322) with increasing level of watering but the addition of Compost and NPK reduced the magnitude of this increase (Figure 7A). The model developed for *U. pumila* data showed a significant increase (*p* < 0.001; F = 15.109) of TRB with the increasing of the level of watering (Figure 7B). When fertilizers were added the magnitude of this increase was lowered in comparison with unfertilized trees (Figure 7B). In particular, TRB for trees fertilized with NPK did not show a significant relationship (*p* = 0.47) with the increasing level of watering (Figure 7B).

#### Root Class to Total Root Biomass Ratio

The highest and the lowest percentage of *P. sibirica* biomass was found for the TR and the FR, which represented, respectively, almost 50 and 3% of the total root biomass (Table 4). Taproot biomass showed the highest increase without both water and fertilizers (i.e., watering only treatment and control watering regime trees), which was almost 25% higher among all the other treatments (Table 4). When NPK was added, TR/TRB did not change with watering regime (Table 4). For Compost addition, TR/TRB was the highest only with the 4 L h^−1^ watering regime. FR/TRB was the lowest for controls for watering only trees, and did not change across watering regimes and fertilizers (Table 4). The same pattern was observed for SR and MR to TRB ratio with unremarkable changes across different watering regimes and fertilization types (Table 3). LR/TRB for watered only trees did not differ between control, 2, and 4 L h^−1^; although it was 55.9% significantly lower for trees watered with 8 L h^−1^ (Table 4). LR/TRB did not differ among watering regimes in the case of NPK addition, while it was the highest in the control compared to watered trees when Compost was added (Table 4). VLR/TRB for watered only trees was the lowest for controls while no differences were detected between watering regimes (Table 4). When NPK was added, the highest VLR/TRB value was found for 4 L h^−1^ watering regime, although not significantly different from the 8 L h^−1^ treatment. The same pattern was found when Compost was added with the highest VLR/TRB value for trees watered with 2 and 8 L h^−1^ (Table 4).

**Table 4:** Ratio between biomass of a specific root class (TR, FR, SR, MR, LR, and VLR) and the biomass of the total root system (TRB) according to two studied plant species (*U. pumila* and *P. sibirica*), management techniques (watering only, watering + NPK, and watering + Compost), and different watering regimes.

**Plant species**	**Management technique**	**Watering regimes**	**% TR/TRB**	**% FR/TRB**	**% SR/TRB**	**% MR/TRB**	**% LR/TRB**	**% VLR/TRB**
*U. pumila*	Watering only	Control	43.5 ± 6^abc^	3.7 ± 1.9^a^	12.7 ± 6.4^a^	19.4 ± 12.1^ab^	16 ± 13.2^ab^	4.7 ± 7.3^a^
		2 L h^−1^	41.1 ± 5.7^abc^	5.2 ± 5.8^a^	12.1 ± 3.9^a^	15.6 ± 7.7^ab^	20.7 ± 10.4^a^	5.3 ± 8.5^a^
		4 L h^−1^	38 ± 6.4^c^	3.3 ± 1.5^a^	9.4 ± 5.4^a^	12.8 ± 9.7^b^	17.3 ± 8.1^a^	17.7 ± 16^a^
		8 L h^−1^	37.2 ± 5.7^c^	2.9 ± 1.3^a^	12.4 ± 4.2^a^	7 ± 5.4^b^	22 ± 13.3^a^	18.4 ± 16.8^a^
	Watering and NPK	Control	45.2 ± 10.1^abc^	4.5 ± 3^a^	8.6 ± 4.4^a^	26.5 ± 11.5^a^	6.4 ± 9.5^ab^	12.1 ± 16.4^a^
		2 L h^−1^	48.5 ± 6.1^ab^	3.5 ± 0.7^a^	9.7 ± 3.9^a^	9.1 ± 4.1^b^	21.7 ± 5.5^a^	7.4 ± 12.3^a^
		4 L h^−1^	50 ± 8.9^a^	3.3 ± 1.5^a^	9.4 ± 5.4^a^	19.3 ± 14.4^ab^	12.2 ± 15.3^ab^	5.8 ± 9^a^
		8 L h^−1^	47.3 ± 7.2^abc^	2.6 ± 1^a^	8.9 ± 3.7^a^	9.5 ± 7.7^b^	14.9 ± 7.9^ab^	16.9 ± 11.6^a^
	Watering and compost	Control	39.8 ± 8.2^bc^	3 ± 2.9^a^	9.8 ± 3.9^a^	26.5 ± 17^a^	11.8 ± 13.2^ab^	9 ± 14.8^a^
		2 L h^−1^	42.6 ± 8.8^abc^	3.1 ± 0.7^a^	7.9 ± 2.9^a^	6.8 ± 2.6^b^	20.7 ± 10.1^a^	19 ± 12^a^
		4 L h^−1^	41.7 ± 9^abc^	4 ± 2.6^a^	13.8 ± 5.1^a^	10.2 ± 5.3^ab^	21.2 ± 9.1^a^	9.1 ± 7.6^a^
		8 L h^−1^	48.6 ± 6.5^ab^	5.3 ± 2.7^a^	9 ± 1.8^a^	16.3 ± 11.4^ab^	12 ± 10.6^ab^	8.8 ± 14.1^a^
*P. sibirica*	Watering only	Control	58.3 ± 9^a^	1.1 ± 0.5^b^	4.2 ± 1.3^a^	7.6 ± 4.7^ab^	22 ± 12.2^b^	6.7 ± 5.7^de^
		2 L h^−1^	44.6 ± 5.8^bcde^	3.7 ± 1.2^a^	5.7 ± 3.2^a^	7.5 ± 2.8^ab^	23.3 ± 10.7^b^	15.1 ± 12.3^cde^
		4 L h^−1^	48.2 ± 8.5^bc^	2.3 ± 1.2^ab^	7 ± 5.9^a^	7.6 ± 5.6^ab^	21.7 ± 6.5^b^	13.1 ± 9.4^cde^
		8 L h^−1^	46.8 ± 4.6^bcd^	3.4 ± 2.5^a^	7.9 ± 2.2^a^	7 ± 2.4^b^	9.7 ± 4.7^c^	25.2 ± 7.1^abc^
	Watering and NPK	Control	–	–	–	–	–	–
		2 L h^−1^	46.1 ± 12.3^bcd^	3.5 ± 1.6^a^	6.2 ± 2^a^	9.2 ± 5^ab^	20.2 ± 9.9^bc^	14.8 ± 15.9^cde^
		4 L h^−1^	35.3 ± 5.5^d^	3 ± 0.7^a^	5 ± 2.4^a^	7.6 ± 4.5^ab^	18.1 ± 7.9^bc^	30.9 ± 12.3^ab^
		8 L h^−1^	46.9 ± 5.8^bcd^	2.9 ± 0.7^a^	4.1 ± 1.7^a^	10.6 ± 7.6^ab^	16.3 ± 9.4^bc^	20.1 ± 17.8^abcd^
	Watering and compost	Control	40.9 ± 3.1^cde^	2.4 ± 1.2^ab^	4.7 ± 2.7^a^	14.2 ± 3.8^a^	37.7 ± 5.3^a^	0^e^
		2 L h^−1^	37.2 ± 3.9^de^	2.5 ± 0.8^ab^	4.4 ± 2.3^a^	5.6 ± 4.6^b^	16.3 ± 7.9^bc^	34 ± 10.8^a^
		4 L h^−1^	51.8 ± 8.9^ab^	2.6 ± 1.3^ab^	5.4 ± 3.1^a^	9.1 ± 4.6^ab^	13.6 ± 5^bc^	17.4 ± 9.9^bcd^
		8 L h^−1^	45.5 ± 6^bcd^	3.3 ± 1.5^a^	5.3 ± 3^a^	7.3 ± 4.9^b^	12.1 ± 8.4^bc^	26.5 ± 8.8^abc^

*Letters indicate significant differences (p < 0.05) among factors for each species*.

*TR, taproot; FR, fine root; SR, small root; MR, medium root; LR, large root; VLR, very large root; TRB, total root biomass*.

In *U. pumila* the highest and the lowest percentage of biomass was found for the TR and the FR, which represented, respectively, almost 40 and 3% of the total root biomass (Table 4). TR, FR, and SR to TRB ratio did not show any significant differences between watering regimes and fertilization types (Table 4). MR/TRB did not differ among watering regimes when watering only trees were analyzed. In the case of NPK addition the highest and the lowest MR/TRB value was found, respectively, for control and 8 L h^−1^ watering regimes. Trees treated with Compost did not show significant variation of MR/TRB between watering regimes (Table 4). Finally, LR and VLR to TRB ratio did not show significant differences among fertilization types and watering regimes (Table 4).

#### Categorical Principal Component Analysis (CATPCA) of Root Class to Total Root Biomass Ratio

The first two principal components (PC1 and PC2), accounted for 53.2% (*P. sibirica*; PC1 29.9%, PC2 23.3%) and 49.9% (*U. pumila*; PC1 30.0%, PC2 19.9%) of the data variance.

**Figure 8:**
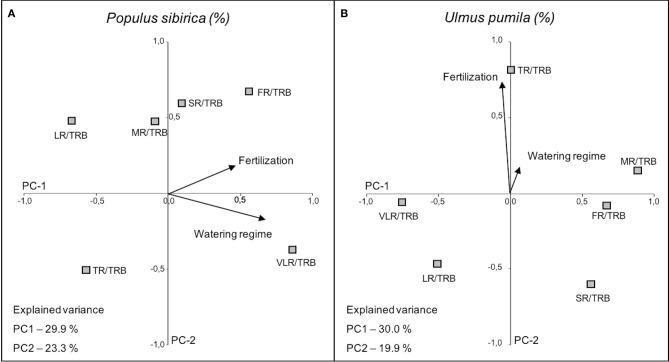
Categorical Principal Component Analysis (CATPCA) biplot of *P. sibirica* **(A)** and *U. pumila* **(B)** indicating the percentage values of the ratio between different root classes and total root biomass projection in relation with watering regime and fertilization. Tightly clustered variables are positively related, while variables at different ends of the figure are negatively related. Arrows represents the mean influence magnitude for each factor.

In the case of *P. sibirica* both fertilization and watering regimes influenced the ratio of root class with total root biomass. In particular, VLR/TRB increased with the increase of watering level (PC-1 axis) and FR/TRB increased with fertilization (PC-2 axis), although the relationship is weaker (Figure 8; Table 4). Moreover, TR/TRB showed an inverse relationship with fertilization while LR/TRB had an inverse relationship with watering regime.

The ratio of biomass of each root class and the total root biomass in *U. pumila* was scarcely influenced by the watering regimes (Figure 8). On the contrary, fertilization affected the biomass ratio (PC-2 axis) and in particular TR/TRB showed a strong increasing relationship with the addition of fertilizers, while SR and LR decreased with fertilization, although less significantly (Figure 8; Table 4). FR, MR, and VLR to TRB ratio were slightly lying on PC-1 axis and they were independent of the treatments.

## Discussion

In the present study, *P. sibirica* and *U. pumila* species were differentially influenced by both watering regime and fertilization. *P. sibirica* root biomass was more influenced by the watering than *U. pumila*, which on the contrary was influenced to a greater extent by fertilization. These findings are in line with previous work dealing with aboveground morpho-physiological traits (Cho et al., 2019), which highlighted that *P. sibirica* in water deficit conditions has a higher water requirement than *U. pumila*.

The explanation for this difference may be related to the biological characteristics of the two species. Indeed, although *Populus* species are fast-growing requiring a relatively higher water supply (Rhodenbaugh and Pallardy, 1993; Isebrands and Richardson, 2014; González et al., 2016), *P. sibirica* is a drought-resistant species adapted to drier and warmer conditions (Tsogtbaatar, 2004; Pearce et al., 2006). On the other hand, *U. pumila* was found to adopt morpho-physiological plasticity mechanisms (e.g., reduced leaf size and increased leaf thickness) to counteract arid environmental conditions (Park et al., 2016; Qin et al., 2016).

For both species analyzed, the root biomass increased together with the increase of watering levels, independently of the diameter class considered. This has been related to the lowered photosynthetic CO_2_ assimilation as the water deficit increased (Tezara et al., 2002; Li et al., 2011; Xu et al., 2015). In particular, for most of the root classes, biomass was remarkably lower in unwatered trees compared with watered trees, while no significant difference was detected between trees grown with 4 and 8 L h^−1^ watering regimes.

Our model highlighted that although root biomass increased with the increasing level of watering, this increment seemed to be lowered when root biomass was analyzed for trees grown with fertilizers. Interestingly, for *P. sibirica*, root biomass did not differ between fertilized and unfertilized trees when no watering and a 2 L h^−1^ regime were considered. At higher levels of watering, the addition of fertilizers seemed to induce a reduction of root growth. In the case of *U. pumila*, this reduction in root growth when fertilizers were applied was already detectable with the 2 L h^−1^ watering regime and was even of greater magnitude with higher levels of watering.

This clear reduction of root growth may be related to both the rate and time of application as well as to the type of fertilizer used in our study, which are important factors for meeting plant needs particularly in dry/arid lands (Abubaker et al., 2020).

Maturity and stability are important parameters for quality assessment of organic fertilizers (Gómez et al., 2006), which refers to the degree of decomposition of phytotoxic organic substances produced during the active composting phase and to the absence of pathogens (Wu et al., 2000). The sheep manure compost that was used in the present work might be compared to unmatured compost characterized by a high concentration of ammonium, organic acids, and other compounds that can be phytotoxic and inhibit root growth (Zucconi et al., 1981b; Chanyasak et al., 1983a,b; Wong, 1985). Although these chemicals do not induce lasting toxic effects in the environment (Zucconi et al., 1981a), long-term effects can occur from unstabilized organic material due to ammonium immobilization by soil microorganism, which makes it unavailable for plant utilization resulting in deficiency problems (Bengston and Cornette, 1973; Terman et al., 1973; Busby et al., 2007; Alburquerque et al., 2012; Abubaker et al., 2015). Furthermore, the low performance of digested manure might be due to the increased concentration of NH${}_{4}^{+}$ (Risberg et al., 2017), which can be lost to the atmosphere by volatilization especially from sandy soil (Ni et al., 2012) and has been generally related to a decrease of root growth (Zhang et al., 2017), or more specifically to the inhibition of the primary root elongation, root biomass (Tian et al., 2008; Giehl and von Wiren, 2014; Morris et al., 2017; Abubaker et al., 2020), and deep roots development (Comfort et al., 1988).

Similarly, the reduction of root growth measured in plants grown with NPK addition might be related to the high application rate, which led to a large quantity of urea-based fertilizer considered phytotoxic (Chen et al., 2020). Also, high N fertilizer application rates to such an early growth stage of seedlings, may induce a growth priority to the aboveground structure, thereby repressing root growth without affecting shoot biomass (Dong et al., 2008; Rogato et al., 2010; Zhang et al., 2017).

These hypotheses to account for the lower root growth are further supported by the findings of soil analysis showing that after 10 years the nitrogen concentration of fertilized soils was almost double that of unfertilized soil, independently of the watering regime considered. Also, the level of nitrogen for the fertilized soil measured within the afforested site was similar to values measured outside. These results suggest a depletion of nitrogen attributable to the tree cover compared to grass and herbaceous cover, which was probably off-set by the fertilization. It is likely that fertilization has balanced the loss of nitrogen that would have occurred due to the tree growth. In our work, however, this demand seems to be different for the two species analyzed. *U. pumila* soil had slightly lower values of nitrate than *P. sibirica* independent of the application of fertilizers and the level of watering. This is in line with previous works (Van der Salm et al., 2006; Wei et al., 2010) that found differences in soil nitrogen across tree species used to afforest former grassland or agricultural lands. Together with the consumption of nitrogen due to the change in land cover, nitrogen leaching might be jointly responsible for low values found under canopy cover in our study for unfertilized soil. Indeed, Callesen et al. (1999) showed that the N leached from newly established forests on previously agricultural land may be higher than those from old-growth forests. Leaching of nitrate is a risk when the availability of inorganic N exceeds the demand from plants and microorganisms (Aber et al., 1989; Gundersen, 1991), and, as in our study, may be induced by the increased input of N due to fertilization (Rosenqvist et al., 2007). This mechanisms has been found to be most severe in sandy soils, similar to the soil type of our study site, that usually have a low retention of nutrients and 20–80% of applied nutrients or chemicals leach or runoff to ground and surface waters (Campbell et al., 1985; Sims et al., 1998; Manevski et al., 2015; Matichenkova et al., 2020).

Both *P. sibirica* and *U. pumila* trees showed the highest biomass investment in the taproot that corresponded, respectively, to almost 50 and 40% of the total root biomass. It is important to underline that since the *P. sibirica* trees were produced from cuttings the taproot we are referring to does not correspond to the seminal root but to the main root axis that may have replaced the pivotal role of the taproot. Indeed, Yang et al. (2014, 2016) found that the taproot contributed the most to anchorage strength, thus it is reasonable that our studied species have allocated the greatest root biomass to the taproot to ensure their stability. Furthermore, our biomass values are in line with values reported in literature that found from 50 to over 75% of belowground biomass allocated to the taproot (Kinerson et al., 1977; Van Lear and Kapeluck, 1995; Laclau, 2003; Miller et al., 2006). In addition, both species showed the lowest biomass values for the fine root category (d < 2 mm), accounting for the 3% of the total root biomass. This finding is in line with general agreement that fine roots rarely represent more than 5% of total tree biomass, although their annual production amounts to 33–67% of the total annual net primary production in most ecosystems (Matamala et al., 2003; McCormack et al., 2015; Montagnoli et al., 2018). Furthermore, *U. pumila* trees allocated more biomass in roots with small and medium diameters (SR and MR) whereas *P. sibirica* allocated more biomass in roots with large and very large diameter (LR and VLR).

These patterns in biomass allocation partitioning across the different root diameter classes changed when management practices were considered. Indeed, since the soil is spatially and temporally highly heterogeneous as a result of the soil type but also of different management practices (Mangalassery et al., 2014), plant roots may regulate their architecture (i.e., growth and branching) in response to soil environmental factors such as water, temperature, mechanical forces, and nutrient availability in addition to genetically determined developmental programs (Montagnoli et al., 2012, 2014, 2019a, 2021; Morris et al., 2017; Dumroese et al., 2019). This highly adaptable behavior, termed developmental plasticity, has been a major determinant for the success of land plants (Wilkinson, 2000; Hodge, 2004). Therefore, the different allocation of biomass among all components of a root system, further modified in relation to management practices, could be considered as a homeostatic response to factors that most limit plant growth (Bloom et al., 1985).

Our data suggest that the two species used different biomass partitioning strategies to face changes in soil characteristics due to the watering regimes and fertilization. In particular, *P. sibirica* increased the VLR components when levels of watering increased. This might be related to the need of *P. sibirica* to enhance the function of water transport from the roots to the stem as water availability increased. Moreover, enlarging the VLR fraction would probably be related to the enhancement of the root mechanical component (i.e., main first order lateral coarse roots) that has to sustain an outgrown tree structure (Dumroese et al., 2019; Montagnoli et al., 2019b, 2020). On the other hand, fertilization was found to increase the FR component suggesting a probable increase of the fine root web to face the higher availability of nutrients that should have occurred once fertilizers were applied (Ahlström et al., 1988; Wang et al., 2019). *U. pumila* did not show noticeable variations in biomass partitioning among different root classes in relation with the increase of levels of watering. This is in accordance with the lower sensitivity of *U. pumila* to the watering levels then *P. sibirica*. However, when fertilizers were applied *U. pumila* trees increased the TR component, reducing the SR and LR components. This might be related to the taproot accumulation function of N-reserves and vegetative storage protein (Noquet et al., 2001; Meuriot et al., 2003) that could be enhanced due to the higher nutrient availability.

Finally, in the present study there was a lack of influence of fertilization on soil organic matter. This could be due to the texture of the soil (70% sand and about 10% clay) and to the relatively low organic enrichment caused by a slow humification. Various authors have observed that organic carbon content and stabilization in the soil increase with increasing clay content (Ali et al., 1966; Ladd et al., 1977; Merckx et al., 1985; Feller and Beare, 1997; Six et al., 2002; Manna et al., 2005; Banger et al., 2009) since it provides protection to soil organic matter and makes it relatively inaccessible through aggregation against microbial and enzyme attack (Mtambanengwe et al., 2004; Strong et al., 2004; Banger et al., 2009). Moreover, in sand textured soils, decomposition of animal manure used in our study is faster than in fine textured soils (van Veen and Kuikman, 1990; Strong et al., 1999; Six et al., 2002), resulting in lower organic carbon (Bossuyt et al., 2001; Six et al., 2001), and short-time soil organic matter accumulation (Chivenge et al., 2011).

## Conclusions

Although soil chemical characteristics were not enhanced over the long-term by the addition of fertilizers, our findings highlighted that increasing the levels of watering directly increased the root biomass development for both *U. pumila* and *P. sibirica*. However, this increment did not significantly differ when 4 and 8 L h^−1^ levels of watering were compared suggesting that an irrigation level of 4 L h^−1^ would yield high root biomass development with low levels of water consumption, and could be recommended for economical, sustainable, and effective reforestation in the semi-arid steppe of Mongolia. Our findings highlighted a possible reduction of root growth occurring when fertilizers were applied. This is unfortunate and might be due to the interplay of type and dose of fertilizers together with the sandy soil that characterized the study area. We therefore strongly suggest to implement afforestation intervention with an *a priori* analysis of the combination of type, dose, and chemistry of fertilizers, soil type, and plant needs for future application. Also, the two species showed a different root biomass developmental pattern with *P. sibirica* being more dependent on the water supply. These findings might be explained by the eco-physiological characteristics of the two species, suggesting that *U. pumila* might be selected for afforesting semi-arid environments with the lowest water consumption. Our data showed the higher and lower root biomass investment, respectively, in the taproot and fine root classes. Finally, the application of different management practices supporting tree growth modified the biomass partitioning across different root diameter classes. Such modification indicated a change in the overall root architecture that has been developed to ensure better tree performance in response to changes in the soil environment.

## Data Availability Statement

The raw data supporting the conclusions of this article will be made available by the authors, without undue reservation.

## Author Contributions

BN-O, DC, and AM conceived the research project. BN-O provided primary funding. AM, BN-O, S-OB, and DC developed the study plan. AM and DC dealt with the methodological approach and equally contributed to data interpretation. S-OB and BN-O were responsible for field excavation and data collection. S-OB and MT performed the data analysis. GSS, BP, and DC provided important insights into the research process. DC prepared the original draft. AM wrote the manuscript and dealt with revision process and finalization. JS and BN-O edited and revised the manuscript. All authors contributed to the article and approved the submitted version.

## Conflict of Interest

The authors declare that the research was conducted in the absence of any commercial or financial relationships that could be construed as a potential conflict of interest.
